# CdTe quantum dots with daunorubicin induce apoptosis of multidrug-resistant human hepatoma HepG2/ADM cells: *in vitro *and *in vivo *evaluation

**DOI:** 10.1186/1556-276X-6-418

**Published:** 2011-06-13

**Authors:** Gen Zhang, Lixin Shi, Matthias Selke, Xuemei Wang

**Affiliations:** 1 State Key Lab of Bioelectronics (Chien-Shiung Wu Lab), Department of Biological Science and Medical Engineering Southeast University, Nanjing, 210096, PR China; 2 Department of Chemistry and Biochemistry, California State University, Los Angeles, CA 90032, USA

## Abstract

Cadmium telluride quantum dots (Cdte QDs) have received significant attention in biomedical research because of their potential in disease diagnosis and drug delivery. In this study, we have investigated the interaction mechanism and synergistic effect of 3-mercaptopropionic acid-capped Cdte QDs with the anti-cancer drug daunorubicin (DNR) on the induction of apoptosis using drug-resistant human hepatoma HepG2/ADM cells. Electrochemical assay revealed that Cdte QDs readily facilitated the uptake of the DNR into HepG2/ADM cells. Apoptotic staining, DNA fragmentation, and flow cytometry analysis further demonstrated that compared with Cdte QDs or DNR treatment alone, the apoptosis rate increased after the treatment of Cdte QDs together with DNR in HepG2/ADM cells. We observed that Cdte QDs treatment could reduce the effect of P-glycoprotein while the treatment of Cdte QDs together with DNR can clearly activate apoptosis-related caspases protein expression in HepG2/ADM cells. Moreover, our *in vivo *study indicated that the treatment of Cdte QDs together with DNR effectively inhibited the human hepatoma HepG2/ADM nude mice tumor growth. The increased cell apoptosis rate was closely correlated with the enhanced inhibition of tumor growth in the studied animals. Thus, Cdte QDs combined with DNR may serve as a possible alternative for targeted therapeutic approaches for some cancer treatments.

## Introduction

Multidrug resistance, a phenomenon of resistance of cancer cells to structurally diverse and mechanically unrelated anti-cancer drugs, is a major obstacle to successful cancer chemotherapy [1]. Cancer cells are different in their sensitivity and response upon treatment with anti-cancer drugs [2]. Anti-cancer drugs have little activity and produce a low percentage of response percentage to treatment with drug-resistant cells. Over-expression of P-glycoprotein (P-gp) is the most frequent event causing multidrug resistance [3]. CdTe quantum dots (Cdte QDs) have primarily received attentions in biological and biomedical fields due to their high luminescence efficiency, photostability, and broad absorption and narrow emission spectra [4]. They have also attracted considerable interest because they exert tumor-inhibiting effects by a mode of action different from other organic compounds [5]. Potential biologically active Cdte QDs have been extensively involved in potential new-type drug design because of their more specific properties.

Liver cancer is one of the most common tumors worldwide and a primary malignancy of the liver. HepG2 cell line has been widely used as the human hepatoma model cell line in the development of new anti-tumor medicines [6]. The classical Topo II inhibitor daunorubicin (DNR) is known as one of the most effective anti-cancer drugs on the market today [7]. Its anti-tumor activity has been reported in clinical trials against a wide variety of tumors. One of the biggest shortcomings of this drug, however, is its low anti-tumor activity against drug-resistant cells, for example adriamycin-resistant human hepatoma HepG2 cells.

Cdte QDs possess good biocompatibility and low toxicity; some recent observations illustrate that Cdte QDs with DNR treatment may indeed lead to improved selectivity toward leukemia cancer cells and facilitate inhibition of the proliferation of targeted cells. Binding the positively charged DNR molecule to a negatively charged surface of Cdte QDs may enhance drug uptake. In this study, we report the biological effects of Cdte QDs capped with negatively charged surface stabilizers (i.e., capped with 3-mercaptopropionic acid) alone or combined with anti-cancer drug DNR treating adriamycin-resistant human hepatoma HepG2 cells, as well as nude mice as model animal systems. We found that Cdte QDs greatly increased the DNR sensitivity against cancer cells. The *in vivo *study also revealed that Cdte QDs with DNR showed a good activity to inhibit tumor growth.

Apoptosis is an important biological process in many systems and can be triggered by a variety of stimuli received by the cells [8]. It is well known that apoptosis can be triggered via two principal signaling pathways: the death receptor-mediated extrinsic apoptotic pathway, and the mitochondrion-mediated (cytochrome c, caspase-9) intrinsic apoptotic pathway [9]. Western blotting was used in this study to explore the mechanism of anti-cancer activity after cell treatment by Cdte QDs with DNR. We found cell apoptosis with a rapid induction of cytochrome c, cleaved caspase-9 and caspase-3 activity, and stimulated proteolytic cleavage of poly-(ADP-ribose) polymerase (PARP) activation, which demonstrate that synergistic effects of Cdte QDs with DNR to induce apoptosis can be through mitochondrion-mediated intrinsic apoptotic pathway.

## Experimental section

### Reagents

The drugs DNR and adriamycin were purchased from Sigma-Aldrich (St. Louis, MO, USA). The RPMI 1640 cell culture medium was obtained from Gibco BRL (Grand Island, NY, USA). The fetal calf serum (FCS) was from HyClone (South Logan, UT, USA). Penicillin, streptomycin, 3-(4,5-dimethyl-2-thiazolyl)-2,5-diphenyl-2H-tetrazolium bromide (MTT), acridine orange/ethidium bromide was all purchased from Sigma-Aldrich (St. Louis, MO, USA).

### Preparation of Cdte QDs

Cdte QDs were prepared as described elsewhere [10]. The water-soluble Cdte QDs capped with negatively charged 3-mercaptopropionic acid. The morphology of the Cdte QDs was characterized by JEM-2100 high-resolution transmission electron microscopy (HRTEM). Dynamic light scattering measurement was carried out (ELS-8000L, Otsuka Electronics Co. Ltd., Osaka, Japan). Emission spectra of the Cdte QDs were measured by a Hitachi-7000 fluorescent spectrometer.

### Cell culture and development of multidrug resistance

Human hepatoma HepG2 cells were purchased from the Institute of Hematology of Tianjin, Chinese Academy of Medical Sciences (Tianjin, China). To develop the drug-resistant cell line (HepG2/ADM), adriamycin was added to HepG2 cells in a stepwise increasing concentration, from 0.05 to 2 μg/ml over 8 months described [11]. Western blotting was used to assess the MDR1 levels of HepG2 and HepG2/ADM cells. The drug-resistant HepG2/ADM cells were cultured in the cell culture medium containing 1 μg/mL adriamycin (Sigma). Both cell lines were maintained in RPMI-1640 medium containing 10% FCS, 100 U/ml of penicillin, and 100 μg/ml of streptomycin at 37°C with 5% CO2.

### Cytotoxicity assays (MTT assay)

HepG2/ADM Cells (2 × 10^3^/well) were plated in 96-well plates. After overnight incubation, HepG2/ADM cells were treated with various concentrations of DNR and various concentrations of Cdte QDs, or 4 μM Cdte QDs with various concentrations of DNR, respectively. After cells were treated for 36 h, 20 μL MTT solution (5 mg/ml) was added to each well. After 4-h incubation, the supernatant was removed and 100 μL DMSO was added to each well. Samples were then shaken for 15 min. The optical density (OD) was read at the wavelength of 540 nm. All experiments were performed in triplicates. Relative inhibition of cell growth was expressed as follows: Percentage (%) = (1 - [OD]test/[OD]control) × 100%.

### Fluorescence microscopic studies

HepG2/ADM cells were treated with 4 × 10^-6 ^mol/L of DNR, 4 μM Cdte QDs + 4 × 10^-6 ^mol/L DNR. Untreated were taken as controls. All samples were maintained for 2 h at 37°C. The fluorescence was captured by IX71 inverted fluorescence microscope (Olympus America Inc., Melville, NY, USA) with the excitation wavelength at 488 nm and emission wavelength at 530 nm.

### Electrochemical analysis of drug uptake

Differential pulse voltammetry was performed on a CHI660b electrochemical workstation to detect the electrochemical response of Cdte QDs and DNR to cells. All measurements were carried out in a three-component electrochemical cell consisting of a glassy carbon electrode as working electrode, a Pt wire as the counter electrode and an Ag wire electrode as the reference electrode. The HepG2/ADM cells were separated from suspension by centrifugation and washed twice; after that, the 1 × 10^6 ^cells were cultured with 4 × 10^-6 ^mol/L DNR, 4 μM Cdte QDs + 4 × 10^-6 ^mol/L DNR in PBS for 2 h at 37°C in a 5% CO2 incubator. The control was treated with PBS.

### Acridine orange/ethidium bromide (AO/EB) staining to detect apoptosis

HepG2/ADM cells were incubated with Cdte QDs + DNR for 48 h. To stain apoptotic cells, the cells were trypsinized for 5 min before adding l μl of AO/EB dye mixture (100 μg/ml acridine orange and 100 μg/ml ethidium bromide) to each well. Cells were viewed under the fluorescent light microscope.

### Flow cytometry analysis

Cells were seeded in 12-well plates at 1 × 10^5 ^cells/well. After incubation for 72 h at 37°C, 5% CO2, HepG2/ADM cells were treated with relative DNR, Cdte QDs, or Cdte QDs + DNR for 48 h. "Annexin-V-FITC apoptosis detection kit" (Keygen, Biotech Co., Ltd, Nanjing, China) was used to determine apoptosis. Flow cytometric analysis was conducted using a BD FACSCanto flow cytometer (BD Biosciences, Franklin Lakes, NJ, USA).

### DNA fragmentation assay

HepG2/ADM cells were incubated with DNR, Cdte QDs, or Cdte QDs + DNR for 72 h, respectively. The untreated cells served as controls. DNA was extracted from HepG2/ADM cells using Apoptotic DNA ladder isolation kit (YuanPingHao Biotechnology Co., Ltd, Beijing, China), and then loaded onto 1% agarose gel. The DNA ladders stained with ethidium bromide were visualized under UV light.

### Immunofluorescence microscopy

After Cdte QDs + DNR treatments, HepG2/ADM cells were washed with PBS and fixed in 100% methanol for 10 min. Cell monolayers were blocked in 5% BSA in PBS for 45 min and incubated for 1 h at room temperature with P-gp antibodies (Invitrogen, Beijing, China), followed by incubation for 1 h with secondary antibodies. The fluorescence was captured by an IX71 inverted fluorescence microscope (Olympus)

### Western blotting analysis *in vitro*

HepG2/ADM cells (1 × 10^5^/well) were plated in 2 mL medium/well in six-well plates. After 72-h treatment of relevant DNR, Cdte QDs, or Cdte QDs + DNR, HepG2/ADM cells lysates were prepared from treatment using modified RIPA lysis buffer. The lysates were subjected to SDS-PAGE/Western blot analysis. The following antibodies were used: anti-cytochrome c, anti-cleaved caspase-9, anti-cleaved caspase-3, PARP (cell signaling, China), GAPDH levels were measured to ensure equal loading of protein. To determine if Cdte QDs + DNR reduced HepG2/ADM cells over-expression P-gp, after 72-h treatment of Cdte QDs + DNR, anti-P-gp antibody was used too.

### Experimental animals

Nude mice were provided by the Animal Feeding Farm of National Institute for the Control of Pharmaceutical and Biological Products (People's Republic of China). All mice were housed in the animal facility and animal experiments were conducted following the guidelines of the Animal Research Ethics Board of Southeast University. HepG2/ADM cells (4-5 × 10^6^) were suspended in 100 μL of culture medium and subcutaneously inoculated into the right flank of mice using a 1.0 mL syringe.

### Intravenous injection of reagents and tumor growth inhibition study

The nude mice inoculated with HepG2/ADM cells were divided into four groups with seven mice in each group: (1) control; (2) DNR; (3) Cdte QDs; (4) Cdte QDs + DNR. When the tumor volume became around 50 mm^3 ^after 1 week of inoculation, treatment was injected for each group. Injection was intravenously administered by tail vein at day 0, 2, 4, 6, 8, 10, 12, 14, 16, and 18. The tumor volume of nude mice were measured and calculated at the 20th days after treatment. The tumor volume calculation was performed using the formula *V *= π/6 × [(*a *+ *b*)/2]^3^, where *a *is the largest and *b *is the smallest diameter of the tumor.

### *In situ *apoptosis by TUNEL staining

Apoptotic cell death in deparaffinized tumor tissue sections was detected using terminal deoxynucleotidyl transferase-mediated dUTP nick end-labeling (TUNEL) with the Klenow DNA fragmentation detection kit (Roche, Indianapolis, IN, USA). Sections were permeabilized with 20 μg/mL protease K, and endogenous peroxidase was inactivated by 3% H2O2 in methanol. Apoptosis was detected by labeling the 3'-OH ends of the fragmented DNA with biotin-dNTP using Klenow at 37°C for 1.5 h. The tumor slides were then incubated with streptavidin horseradish peroxidase conjugate, followed by incubation with 3,3'-diaminobenzidine and H2O2. Apoptotic cells were identified by the dark brown nuclei observed under light microscope.

### Statistical analysis

Results were presented as mean ± SD. A *t *test was performed in each group for each time point. A value of *p *< 0.05 was considered statistically significant.

## Results and discussion

### Results

#### Characterization of CdTe quantum dots

**Figure 1:**
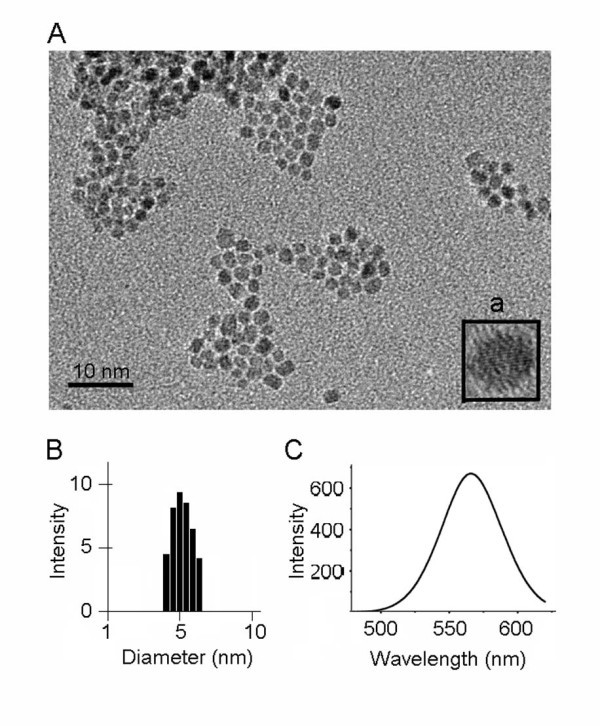
**TEM images of Cdte QDs**: (**A**) the low magnification images Cdte QDs, (a) HRTEM image of an individual nanocrystal of Cdte QDs. (**B**) Size of Cdte QDs suspended in cell culture medium was analyzed by dynamic light scattering. (**C**) Emission spectrum of Cdte QDs, excitation wavelength at 330 nm.

The water-soluble Cdte QDs capped with negatively charged 3-mercaptopropionic acid were prepared according to the procedure as reported previously. Our TEM study illustrates that the average size of Cdte QDs was about 4 nm, as shown in Figure 1A, and an HRTEM individual nanocrystal of Cdte QDs (Figure 1A a, HRTEM). The Cdte QDs in cell culture medium were about 5 nm, as characterized with dynamic light scattering (Figure 1B). The typical fluorescence spectrum of the Cdte QDs was shown in Figure 1C.

#### Cytotoxicity of Cdte QDs with DNR on HepG2/ADM cells

**Figure 2:**
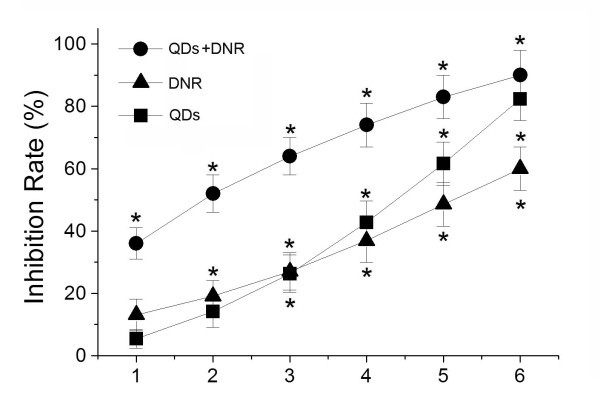
**MTT assay of the growth inhibition rate of HepG2/ADM cells after various cellular treatments**. The HepG2/ADM cells were treated with 1 × 10^-6^, 4 × 10^-6^, 16 × 10^-6^, 64 × 10^-6^, 12.8 × 10^-5^, and 51.2 × 10^-5 ^mol/L of DNR; 1, 2.5, 5, 10, 20, and 40 μM Cdte QDs; or 4 μM Cdte QDs with 1 × 10^-6^, 4 × 10^-6^, 16 × 10^-6^, 64 × 10^-6^, 12.8 × 10^-5^, and 51.2 × 10^-5 ^mol/L of DNR, respectively. *p < 0.05, indicates the significant difference in comparison to no treatment.

The MTT assay was carried out to explore the relative inhibition for the proliferation of the cells. The cells were treated with different concentrations of DNR or Cdte QDs, or treated by different concentrations of DNR combined with Cdte QDs for 36 h. Since HepG2/ADM cells are drug-resistant cell line, the high-concentration DNR treatment only causes low growth inhibition for HepG2/ADM cells (as shown in Figure 2). However, the growth inhibition rate was significantly increased when HepG2/ADM cells were treated by DNR combined with Cdte QDs. Therefore, it is evident that the significant enhancement of the cell proliferation inhibition may be facilitated due to a synergistic effect of Cdte QDs with DNR to the drug-resistant HepG2/ADM cells.

#### Fluorescence microscopy and electrochemical assay of cellular drug uptake

**Figure 3:**
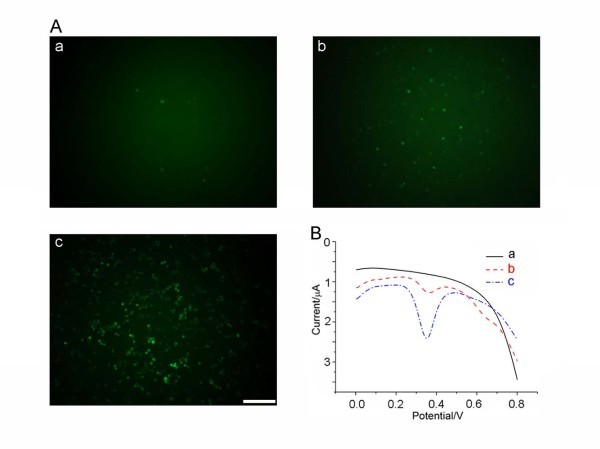
**Measurement of cellular fluorescence and drug uptake**. (**A**) Inverted fluorescence microscopy of HepG2/ADM cells; (a) control, (b) 4 × 10^-6 ^mol/L DNR, and (c) 4 μM Cdte QDs + 4 × 10^-6 ^mol/L DNR; bar, 100 μm. (**B**) Differential pulse voltammetry study of DNR residue outside HepG2/ADM cells after cell treatment for 2 h. (a) PBS; (b) 4 μM Cdte QDs + 4 × 10^-6 ^mol/L DNR treatment and cells for 2 h; and (c) 4 μM DNR. Pulse amplitude, 0.05 V; pulse width, 0.05 s; and pulse period, 0.2 s.

Based on the above study, bio-imaging of DNR in HepG2/ADM cell lines were assayed with inverted fluorescence microscopy. For the control cells without treatment, we observed almost no intracellular fluorescence HepG2/ADM cells (Figure 3A a). DNR treatment showed relatively low fluorescence in HepG2/ADM cells (Figure 3A b). However, the intracellular fluorescence in HepG2/ADM cells increased dramatically upon treatment with DNR bound to the negatively charged surface of QDs (Figure 3A c). To understand the mechanism of this effect, electrochemical study was used to detect the interaction between DNR and HepG2/ADM cells. The results revealed that after treatment by Cdte QDs and DNR for 2 h, the peak current of the DNR residue outside HepG2/ADM cells decreased more significantly than that with DNR treatment alone, suggesting that more significant decrease of the DNR residue outside HepG2/ADM cells occurs with the treatment of Cdte QDs and DNR (Figure 3B). These observations indicate that Cdte QDs could readily facilitate the uptake of the DNR into HepG2/ADM cells.

#### Staining and flow cytometry analysis to detect apoptosis

**Figure 4:**
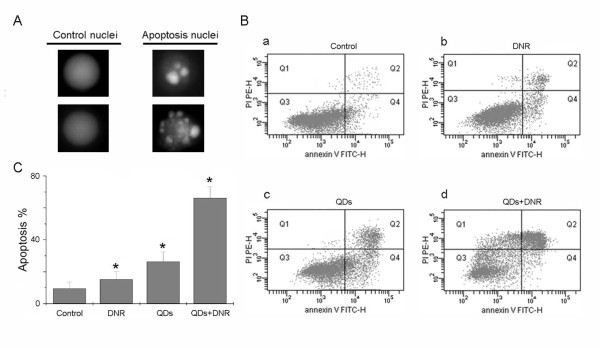
**Assay of cell apoptosis rate and morphological images**: (**A**) Detection of apoptotic and normal cells by acridine orange staining. Control cell nuclei, apoptotic nuclei from HepG2/ADM cells ware observed. (**B**) HepG2/ADM cells detected by flow cytometry using Annexin-V-FITC method. (a) control treatment; (b) 4 × 10^-6 ^mol/L DNR treatment; (c) 4 μM Cdte QDs treatment; and (d) 4 μM Cdte QDs + 4 × 10^-6 ^mol/L DNR for 36 h. (**C**) Quantitative analysis of apoptotic cells after various treatments shown in (B). *p < 0.05, compared to the control treatment.

Using acridine orange/ethidium bromide (AO/EB) dye mixture staining for apoptotic cells, apoptotic nuclei were identified by their distinctively marginated and fragmented appearance under the fluorescence microscope. The apoptotic nuclei of HepG2/ADM cells (Figure 4A, apoptosis nuclei) at 72 h could be identified by their distinctively marginated and fragmented appearance. For the control cells without treatment, cells nuclei were normal as shown in (Figure 4A, control nuclei). Figure 4B shows that Annexin-V-FITC apoptosis detection, Cdte QDs + DNR induced a much higher HepG2/ADM cell apoptosis rate than that of DNR, Cdte QDs, or untreated control. We found that the percentage of apoptotic cells was 67.4%, 26.8%, 15.2%, 8.5% for the treatment with Cdte QDs + DNR, Cdte QDs, DNR, untreatment, respectively (Figure 4C).

#### DNA fragmentation assay

**Figure 5:**
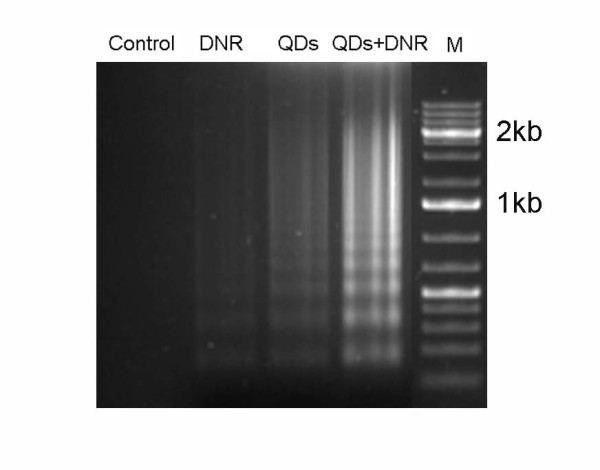
**DNA fragmentation in HepG2/ADM cells after different treatments**. Genomic DNA was isolated from HepG2/ADM cells. DNA ladders were visualized under UV light with ethidium bromide staining. HepG2/ADM cells treated with: control treatment; 4 × 10^-6 ^mol/L DNR; 4 μM Cdte QDs; and 4 μM Cdte QDs + 4 × 10^-6 ^mol/L DNR for 72 h.

The DNA fragmentations were examined. When HepG2/ADM cells were treated with Cdte QDs + DNR, the intensity of fragmented chromosomal DNA bands was much higher than that observed from cells treated with Cdte QDs, or DNR alone (Figure 5). These results provide evidence that the remarkable enhancement of apoptosis was induced by synergistic effects of Cdte QDs and DNR on HepG2/ADM cells.

#### Signal pathway of treatments in HepG2/ADM Cells

**Figure 6:**
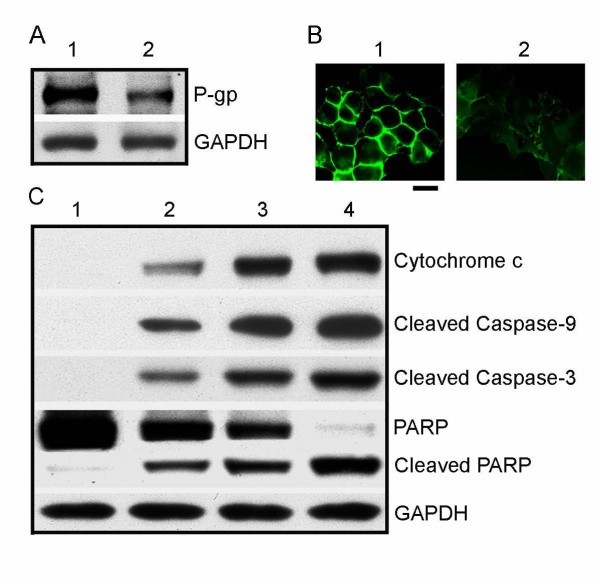
**Signal pathway analysis**. (**A**) Western blotting analysis of P-gp in HepG2/ADM cells. HepG2/ADM cells without treatment were used as control (lane 1). Lysates were prepared from the cells treated 4 μM Cdte QDs with 4 × 10^-6 ^mol/L DNR (lane 2). (**B**) The control cells without any treatment (1). The images were taken from cells treated with 4 μM Cdte QDs with 4 × 10^-6 ^mol/L DNR for 72 h (2). Bar, 20 μm. (**C**) Western blotting analysis of cytochrome c released in HepG2/ADM cells: group 1, control group (lane 1); group 2, 4 × 10^-6 ^mol/L DNR (lane 2); group 3, 4 μM Cdte QDs (lane 3); and group 4, 4 μM Cdte QDs with 4 × 10^-6 ^mol/L DNR (lane 4). The following antibodies were used: anti-cleaved caspase-9, anti-cleaved caspase-3, and anti-PARP antibody. GAPDH was served as a loading control.

Treatment of human HepG2/ADM cells with Cdte QDs + DNR for 72 h caused decrease in the amount of P-gp protein expression compared with control treatment (Figure 6A). Cdte QDs + DNR treated cell monolayers and immunostaining signals of P-gp protein were reduced and disrupted (Figure 6B). To further understand the molecular mechanisms underlying the synergistic effects of Cdte QDs + DNR-mediated apoptosis in HepG2r/ADM cells, we investigated apoptosis-related protein expression in the cells (Figure 6C). DNR or Cdte QDs cannot induce apoptosis strongly in HepG2r/ADM cells due to multidrug resistance. Interestingly, combined treatment of Cdte QDs + DNR strongly caused cytochrome c to be released into the cytosol and significantly activated caspase-9 and caspase-3 and induced degradation of its substrates, PARP. These data suggest that Cdte QDs with DNR treatment involve the release of cytochrome c from the mitochondria, which subsequently causes apoptosis by activation of caspase-9, 3 in HepG2r/ADM cells.

#### Tumor growth inhibition study

**Figure 7:**
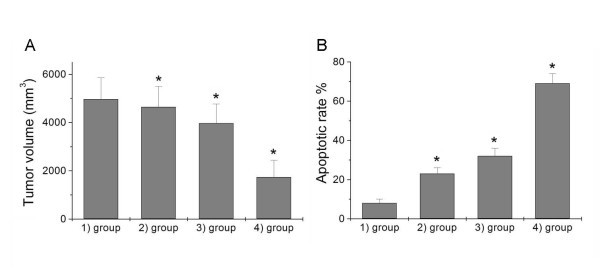
**Inhibition of tumor growth in HepG2/ADM nude mice with different treatments**. **(A) **The different treatment effects on the tumor growth inhibition in nude mice inoculated with HepG2/ADM cells: group 1, no treatment, served as a control group; group 2, 4 × 10^-6^ mol/kg DNR; group 3, 4 μmol/kg Cdte QDs; and group 4, 4 μmol/kg Cdte QDs with 4 × 10^-6^ mol/kg DNR. **(B) **Quantitative analysis of apoptotic cells using TUNEL staining after various treatments. HepG2/ADM xenograft tumors treated as follows: group 1, control group; group 2, 4 × 10^-6^ mol/kg DNR; group 3, 4 μmol/kg Cdte QDs; and group 4, 4 μmol/kg Cdte QDs with 4 × 10^-6^ mol/kg DNR.

The nude mice were inoculated with HepG2/ADM cells and the subsequent tumor growth was recorded after various treatments. From Figure 7A, the HepG2/ADM nude mice, the tumor volume of the control group was enlarged to almost 4970 mm^3 ^(Figure 7A, group 1). Treatment with DNR or Cdte QDs alone has mild inhibitory effect on the tumor growth in the HepG2/ADM mice due to multidrug resistance of the HepG2/ADM cell system (groups 2 and 3, respectively). In the group treated with Cdte QDs + DNR (group 4), tumor growth was significantly inhibited.

#### Analysis of cell apoptosis in HepG2/ADM xenograft tumors

The synergistic effect of Cdte QDs + DNR on the apoptosis induction in the xenograft tumors excised from HepG2/ADM nude mice, the apoptotic rate in the control group was around 8.2% (Figure 7B). Cdte QDs + DNR treatment causes a striking increase in the number of TUNEL-positive nuclei, compared to DNR or Cdte QDs treatment alone. The result of apoptosis rate was well correlated with the result of tumor growth inhibition in the studied animals.

### Discussion

Clinical efficacy of many anti-cancer drugs is limited by the development of drug resistance [12]. In this study, daunorubicin was not effective against HepG2/ADM tumors. This is in agreement with previous studies, which have shown that HepG2/ADM tumor cells overexpress P-gp, and exhibit multidrug-resistant phenotype. We demonstrated that a combination of Cdte QDs and DNR where the DNR is bound to the Cdte QDs surface by electrostatic interaction will improve the accumulation of daunorubicin in tumor cells. The same or even certain high concentration of DNR did not cause a significant reduction in cell viability in HepG2/ADM cells. However, when HepG2/ADM cells were treated with Cdte QDs and DNR, we observed a remarkable enhancement of cell growth inhibition (Figure 2). The results suggest that the synergistic effect of Cdte QDs with DNR can induce cell growth inhibition of drug-resistant HepG2/ADM cells *in vitro*.

We demonstrate that DNR taken in by cellular behavior with synergistic effect of Cdte QDs was significantly higher than that with only DNR treatment. Over-expression of P-glycoprotein is the most frequent event causing multidrug resistance. With Cdte QDs + DNR treatment, the expression of P-glycoprotein was remarkably reduced when compared with the control treatment. It is already known that nanoparticles can cause the formation of "holes" on the surface of cell membranes, which may increase the permeability of the respective cell membranes and thus facilitate uptake of the anti-cancer drug into cancer cells and enhance drug accumulation in target cells [13]. This may be the two reasons why Cdte QDs + DNR increase the intracellular drug concentration dramatically and thus enhance the inhibition of the proliferation to target drug-resistant cancer cells. Furthermore, Cdte QDs with negatively charged surface may combine with anti-cancer drugs such as DNR which is positively charged through electrostatic interaction.

Two major types of cell death are recognized: apoptosis and necrosis [14]. Apoptosis is a regulated process that can be triggered by different stimuli and is mediated by a cascade of enzymes. Necrosis is a catastrophic form of cell death which does not involve the regulated action of enzymes. Studies have demonstrated that the presence of smaller DNA fragments are believed to reflect the release of nucleosomes from apoptotic cells and higher molecular weight DNA molecules are believed to reflect release from necrotic cells [15]. Apoptosis results in fragmentation of cells into apoptotic bodies which are engulfed by neighboring cells and macrophages [16]. However, uptake of necrotic cells has been reported to be less efficient than phagocytosis of apoptotic cells. So active anti-cancer drugs induce apoptosis in malignant cells should be a main way to clinical anti-tumor. Interestingly, we found that Cdte QDs + DNR can induce drug-resistant HepG2/ADM cell apoptosis rate significantly higher than that of Cdte QDs, or DNR alone treatment *in vitro*. Moreover, we analyzed the cells apoptosis morphology from various assay, nuclei staining. When cells were treated with Cdte QDs + DNR, they exhibited characteristic morphological features of apoptosis, such as chromosomal condensation and DNA fragment. With flow cytometry assay, we analyzed quantitative apoptotic cells after various treatments, the Cdte QDs + DNR could be used as inducing HepG2/ADM cells apoptosis with relatively low concentration.

Apoptosis is a regulated process that can be triggered by different stimuli and is mediated by a cascade of enzymes [17]. The realization of mechanisms will enable optimization of chemotherapy for the treatment of cancer [18]. To further understand the molecular mechanisms underlying the Cdte QDs + DNR treatment-mediated apoptosis in HepG2/ADM cells, we investigated apoptosis-related protein expression in HepG2/ADM cells. Cdte QDs + DNR treatment induces cytochrome c release, causing caspase-9 activation. Cleaved caspase-9 activated caspase-3 that correlated with the increased expression of cleaved PARP after relevant treatments [19,20]. Subsequently, DNA fragmentation is induced during the cells apoptosis by cleaved PARP expression. Compared to Cdte QDs or DNR treatment, Cdte QDs + DNR treatment showed much stronger inducing apoptosis effect.

As the above results illustrated, we recognized the evidence of apoptosis of HepG2/ADM cells *in vitro*. It is possible that Cdte QDs + DNR could play a critical role in inducing apoptosis *in vivo*. The tumor growth in group 4 nude mice (treated with Cdte QDs + DNR) was suppressed most efficiently. Cdte QDs or DNR alone cannot significantly inhibit the tumor growth in HepG2/ADM mice due to multidrug resistance of this cell line. Our present study also shows apoptosis in tumor cells was induced by three kinds of treatment with TUNEL assay. The results of the TUNEL assay are consistent with the tumor growth inhibition results. Our observations indicate that the growth-inhibitory effect of Cdte QDs + DNR treatment is related to its ability to induce apoptosis, as evidenced by TUNEL assay. Taken together, our data support the thesis that Cdte QDs + DNR treatment plays an important role in inducing drug-resistant HepG2/ADM cell apoptosis and tumor suppression, and furthermore suggest that Cdte QDs + DNR treatment therapy might provide a powerful treatment for liver cancer.

## Conclusion

In summary, in this study, we have investigated the interaction mechanism and synergistic effect of 3-mercaptopropionic acid-capped Cdte QDs with the anti-cancer drug DNR on the induction of apoptosis of drug-resistant human hepatoma HepG2/ADM cells. Our observations demonstrate that Cdte QDs readily facilitated the uptake of the DNR into HepG2/ADM cells by electrochemical assay. Apoptotic staining, DNA fragmentation, and flow cytometry analysis further demonstrate that treatment of Cdte QDs together with DNR can clearly activate apoptosis in HepG2/ADM cells. Cdte QDs + DNR treatment activated caspases protein expression. While the Cdte QDs + DNR treatment could reduce the effect of P-glycoprotein (P-gp). Moreover, our *in vivo *study indicates that the treatment of Cdte QDs together with DNR effectively inhibited the human hepatoma HepG2/ADM nude mice tumor growth. The increased cell apoptosis rate was closely correlated with the enhanced inhibition of tumor growth in the studied animals. Thus, Cdte QDs combined with DNR may serve as a new effective additive agent to overcome the drug resistance and thus as a novel strategy to sensitively track the respective cancer cells for efficient cancer chemotherapy.

## Competing interests

The authors declare that they have no competing interests.

## Authors' contributions

Respond: GZ carried out the cell biology and molecular studies. LS prepared the Cdte QDs. MS participated in the design of the study. XW conceived of the study, and participated in its design and coordination. All authors read and approved the final manuscript.
